# Perventricular VSD closure to decrease surgical complexity in a developing congenital heart surgery program

**DOI:** 10.1186/1749-8090-8-S1-P139

**Published:** 2013-09-11

**Authors:** J Breinholt, I Polivenok, O Buchneva, M Gelatt, M Cardarelli

**Affiliations:** 1 Pediatric Cardiology, International Children's Heart Foundation, Memphis, TN, USA; 2 Cardiac Surgery and Emergent Cardiology, Institute of General and Urgent Surgery, Kharkov, Ukraine; 3 Congenital Cardiac Surgery, International Children's Heart Foundation, Memphis, TN, USA

## Background

Hybrid procedures are increasingly employed in established pediatric cardiology and cardiac surgery programs. Utilization in developing programs has not been reported.

## Methods

Case report of an infant who underwent “hybrid” closure of 2 muscular VSDs (mVSD) in a developing congenital heart surgery program.

## Results

A 4 month old with 2 mVSDs and prior pulmonary artery band placement was recommended for definitive repair as part of a training mission in a developing congenital heart surgery program. Due to patient size and VSD location, surgery or catheterization were considered a significant challenge for a developing program. Perventricular VSD closure, a novel technique typically utilized in well-established programs, was chosen. After sternotomy, the surgeon inserted a needle in the right ventricular free wall and a guidewire was advanced across one VSD. Lacking transesophageal echocardiographic capabilities, transthoracic echocardiography was used for guidance. The needle was exchanged for an introducer sheath. This procedure was repeated with the second VSD. After introducer sheaths were positioned across the VSDs, the interventional cardiologist deployed a 6mm and an 8 mm Amplatzer mVSD occluders in rapid sequence. After echocardiographic confirmation, the devices were simultaneously released. The pulmonary band was removed without extracorporeal circulation. The infant was extubated within 48 hours and had an uneventful postoperative course.

## Conclusion

Collaborative procedures involving surgeons and interventional cardiologists provide the means to reduce procedural complexity and morbidity. While “hybrid” procedures are largely performed in well-established programs, they provide an attractive management alternative in developing programs, potentially reducing the need for cardiopulmonary bypass and recovery times. Under specific circumstances, transthoracic echocardiography can provide quality guidance similar to transesophageal approach.

